# Supplementary material for: Bronchoscopic Features and Morphology of Endobronchial Tuberculosis: A Malaysian Tertiary Hospital Experience
Source: J Clin Med. 2022 Jan 28;11(3):676. doi: 10.3390/jcm11030676 (PMC8836898; doi:10.3390/jcm11030676)
Supplement: Supplementary file 1 [file jcm-11-00676-s001.zip › jcm-1508598-SI.pdf]

Case	Age	Sex	Comorbidity factors	Symptoms	Most obvious findings on CXR	CXR localization	Indication for bronchoscopy
1	25	F	None	Cough, fever, LOW	consolidation	RML	(-)PTB
2	16	F	None	Cough, fever, LOW, haemoptysis	consolidation	RUL	(-)PTB
3	67	M	DMT2	Cough, fever	consolidation	RUL	(-)PTB
4	55	F	None	Cough, fever, LOW	consolidation	RML, RLL	(-)PTB
5	52	F	None	Cough	consolidation	RUL	(-)PTB
6	48	F	None	Cough, LOW	consolidation + cavitations	LUL	(-)PTB
7	61	M	DMT2 + metastatic rectal cancer	LOW + Incidental findings of RUL cavitation on rectal cancer staging	consolidation + cavitations	RUL	(-)PTB
8	74	F	DMT2 + ESRD	Cough + LOW + dyspnea + recurrent hypoglycemia	consolidation	RUL + RML + LUL + LML	(-)PTB
9	25	F	DMT2	Cough + haemoptysis	consolidation + cavitation	RUL + RLL + LLL	(-)PTB
10	48	M	Post kidney transplant on steroid and immunosuppressive drugs	Cough + fever	consolidation + Pleural effusion	RUL	(-)PTB
11	38	F	SLE not on steroid	Cough + fever	consolidation	LLL	(-)PTB
12	25	F	None	Cough + fever	consolidation	LUL	(-)PTB
13	52	M	HIV positive	Cough + fever	consolidation + LUL mass	LUL, RML	Persistent consolidation
14	33	M	Cerebral Palsy (quadriplegic)	Cough + fever + LOW + haemoptysis	Lung mass	RLL	(-)PTB

15	29	M	HIV positive	Cough + fever + LOW	consolidation	LUL	Persistent consolidation
16	43	F	None	LOW + right supraclavicular neck swelling	consolidation	RML + RLL	(-)PTB
17	33	F	None	Cough + fever + LOW	Widened mediastinum		(-)PTB

Table S1. Demographics and clinical features of EBTB patients.

Case	Sputum AFB/MTB culture	Lavage for AFB smear/MTB culture	Endobronchial biopsy MTB culture	Bronchoscopic Localization	Bronchoscopic Features (Chung's classification)	Endobronchial biopsy HPE (with AFB stain)
1	-/o	+/+	o	RML	Caseating	Granulomatous inflammation (-)
2	-/+	-/+	o	Trachea + RMB	Granular	Caseating Granulomatous Inflammation (+)
3	-/o	-/+	-	RUL + RLL	Ulcerative	Granulomatous Inflammation (-)
4	-/o	+/+	o	RML + RLL	Tumorous	Chronic Inflammation (+)
5	o/o	-/+	-	RBI	Edematous hyperaemic	Granulomatous inflammation (-)
6	-/+	+/o	+	Carina + LMB	Caseating	Caseating Granulomatous Inflammation (-)
7	o/o	-/+	+	RUL	Edematous hyperaemic	Chronic Inflammation (-)
8	o/o	+/+	o	RUL	Caseating	Caseating Granulomatous Inflammation (+)
9	-/o	-/+	+	RUL	Edematous hyperaemic	Granulomatous inflammation (-)
10	-/+	-/+	o	RLL	Caseating	Granulomatous Inflammation (+)
11	-/-	+/+	+	LML + LLL	Caseating	Caseating Granulomatous Inflammation (+)
12	-/o	+/-	+	LMB	Caseating	Caseating Granulomatous Inflammation (+)
13	-/-	-/-	-	Carina + LMB + RML + LUL + LLL	Granular	Granulomatous Inflammation (-)
14	-/-	-/+	o	RLL	Tumorous	Granulomatous Inflammation (+)
15	-/o	-/-	-	LML	Caseating	Caseating Granulomatous Inflammation (+)
16	-/o	-/-	-	RML	Caseating	Caseating Granulomatous Inflammation (-)
17	-/o	-/-	-	RUL + RLL	Caseating	Caseating Granulomatous Inflammation (-)

Table S2. Bronchoscopic findings and EBTB subtypes.

Case	Duration of anti tuberculosis	Symptoms improvement post TB treatment	Repeat bronchoscopy post TB treatment	Endobronchial lesion findings of repeated bronchosopy after treatment completion
1	6 month	Yes	Yes	Resolved endobronchial lesion
2	6 month	Yes	Yes	Resolved endobronchial lesion
3	9 month	Yes	No	NA
4	6 month	Yes	Yes	Resolved endobronchial lesion
5	6 month	Yes	No	NA
6	6 month	Yes	No	NA
7	7 month	Yes	No	NA
8	2 month	Death after 2 month on anti TB	No	NA
9	9 month	Yes	No	NA
10	12 month	Yes	No	NA
11	6 month	Yes	Yes	Resolved endobronchial lesion
12	6 month	Yes	Yes	Resolved endobronchial lesion
13	6 month	Yes	No	NA
14	9 month	Yes	Yes	Resolved endobronchial lesion
15	9 month	Yes	No	NA
16	6 month	Yes	No	NA
17	6 month	Yes	No	NA

Table S3. Bronchoscopic findings of EBTB patients post treatment.
